# Strong relationship between dyslipidemia and the ectopic ossification of the spinal ligaments

**DOI:** 10.1038/s41598-022-27136-4

**Published:** 2022-12-30

**Authors:** Tsutomu Endo, Masahiko Takahata, Ryo Fujita, Yoshinao Koike, Ryota Suzuki, Yuichi Hasegawa, Toshifumi Murakami, Misaki Ishii, Katsuhisa Yamada, Hideki Sudo, Norimasa Iwasaki

**Affiliations:** 1 Department of Orthopedic Surgery, Hokkaido University Graduate School of Medicine, Kita-15 Nishi-7, Kita-ku, Sapporo, Hokkaido 060-8638 Japan; 2 Department of Orthopedics, Hakodate Central General Hospital, 33-2 Hon-cho, Hakodate, Hokkaido 040-8585 Japan

**Keywords:** Neuroscience, Spine regulation and structure, Risk factors, Health occupations, Orthopaedics

## Abstract

Obesity and metabolic disturbances are prevalent in ossification of the posterior longitudinal ligament (OPLL) and ossification of the ligamentum flavum (OLF); however, the involvement of dyslipidemia (DL) in OPLL/OLF remains uncertain. We investigated the association between dyslipidemia and OPLL/OLF using a dataset of 458 individuals receiving health screening tests, including computed tomography. Subjects were grouped according to the presence or location of OPLL/OLF: controls (no OPLL/OLF, n = 230), OLF (n = 167), cervical OPLL (n = 28), and thoracic OPLL (n = 33). They were also grouped according to the presence of dyslipidemia (DL[+], n = 215; DL[−], n = 243). The proportion of dyslipidemia in the OLF and OPLL groups was 1.6–2.2 times higher than that in the control group. The proportion of OLF and OPLL in the DL(+) group was significantly higher than that in the DL(−) group (OLF, 43% vs. 29%; cervical OPLL, 14.4% vs. 3.2%; thoracic OPLL, 11.1% vs. 3.7%). Multivariate logistic regression analysis showed an association between all ossification types and dyslipidemia. This study demonstrated an association of dyslipidemia with OPLL/OLF; further investigation on the causal relationship between dyslipidemia and ectopic spinal ligament ossification is warranted to develop a therapeutic intervention for OPLL/OLF.

## Introduction

Ossification of the posterior longitudinal ligament (OPLL) and ossification of the ligamentum flavum (OLF) are the major causes of myelopathy in the East Asian population ^1–8^, and are often coexistent ^9–11^. Recent epidemiological studies have reported that obesity is a common aggravating factor in patients with diffuse types of OPLL and OLF, in which the entire spinal ligament tends to ossify ^10–15^. This suggests that lifestyle-related diseases and visceral obesity may be involved in the development and progression of these conditions ^11–13^.

Dyslipidemia is caused by malnutrition, obesity, sarcopenic obesity, and genetic predisposition and is closely related to metabolic disturbances^16–18^. If dyslipidemia is left untreated, atherosclerosis will develop. This may cause ischemic heart disease and stroke, and increase the risk of fatty liver and other diseases^16–19^. Lipid metabolism also regulates osteoblasts via the Wnt signaling pathway and is closely related to calcification mechanisms and bone metabolism^20–22^. In recent years, the association of OPLL/OLF with obesity, malnutrition, genetic factors, and metabolic dysfunction–associated fatty liver disease (MAFLD) has become clear^10–15,23–26^. This suggests that metabolic disturbances related to spinal ligament ossification could affect systemic bone metabolism and trigger ectopic ossification. Despite the common features of dyslipidemia and spinal ligament ossification, no study has investigated their relationship in detail.

To date, there is no effective treatment for OPLL/OLF, and this motivated us to identify the underlying causes associated with the onset and exacerbation of this disease. Hence, we aimed to investigate the association between dyslipidemia and OPLL/OLF using a large dataset of subjects who underwent health screening tests, including computed tomography (CT).

## Methods

### Study design

A retrospective cross-sectional study was conducted in accordance with the Declaration of Helsinki (1964), including subjects between April 2020 and May 2021. The study was approved by ethical review board of the Hakodate Central Hospital and Hokkaido University Hospital, and the need for obtaining patients’ informed consent was waived owing to the retrospective nature of the study and the deidentified data used.

### Patients

**Figure 1:**
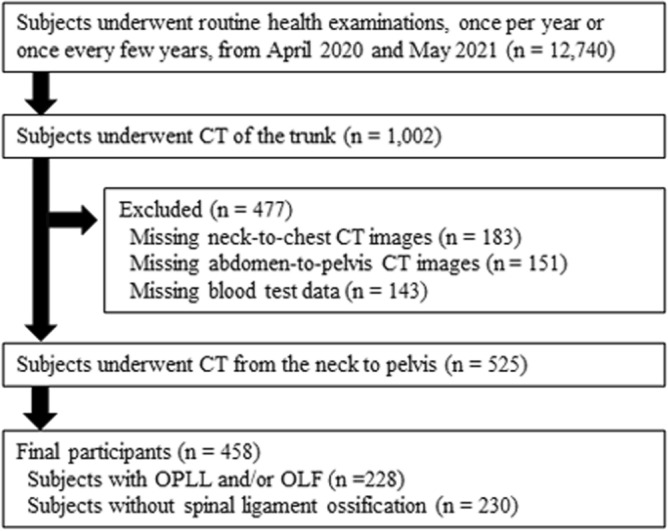
Flow chart of study participants. CT, computed tomography; OLF, ossification of the ligamentum flavum; OPLL, ossification of the posterior longitudinal ligament.

A database of 12,740 Japanese patients from a single institution was used. All subjects, with or without symptoms, underwent routine health examinations once per year or once every few years. Most of the subjects were community residents and facility staff, including physicians, nurses, nursing assistants, therapists, and clerks; the majority underwent blood tests at their discretion. The subjects underwent CT of the trunk at their discretion; they also selected the scan region (neck to chest, abdomen to pelvis, or neck to pelvis). Among the 1,002 subjects who underwent CT, 525 were selected for whom CT allowed assessment of the cervical spine to pelvis region and for whom blood test data were available. Finally, a total of 458 subjects (251 men, 207 women), aged 30 to 78 years, with OLF and/or OPLL and without spinal ligament ossification were included in this study (Fig. 1).

### Demographics and comorbidities

Demographic data were obtained from all subjects using a questionnaire assessing height, weight, smoking history, the presence of comorbidities (hypertension, diabetes mellitus, dyslipidemia, ischemic heart disease, stroke, and renal disease), and the current and past use of medication for the comorbidities. The questionnaire also assessed the presence of weight gain from the age of 20 years and exercise routine of more than 30 min per day.

### Serological assessment parameters

All serological assessments were performed using fasting blood samples. The assessed parameters included total cholesterol (T-Cho), triglycerides (TG), high-density lipoprotein cholesterol (HDL-C), low-density lipoprotein cholesterol (LDL-C), glycated hemoglobin A1c (HbA1c), and blood glucose. The LDL-C/HDL-C ratio (L/H ratio) was calculated and used as one of the parameters to indicate the severity of dyslipidemia. The rationale is based on the suggestion that the L/H ratio is a better predictor of atherosclerosis progression and ischemic cardiovascular disease than LDL-C and HDL-C separately; an L/H ratio ≤ 2.0 significantly inhibits the progression of coronary artery plaque, while a ratio ≤ 1.5 further strengthens this effect^27^.

### Diagnostic criteria for dyslipidemia by the Japan Atherosclerosis Society

For adults, the 2012 guidelines of the Japan Atherosclerosis Society define lipid abnormality as a TG concentration ≥ 150 mg/dL, LDL-C ≥ 140 mg/dL, and/or HDL-C < 40 mg/dL^28^.

In the present study, subjects with dyslipidemia were defined as those who met any of the above criteria or who were taking therapeutic drugs for dyslipidemia.

### Assessment of the presence of OLF and OPLL

**Figure 2:**
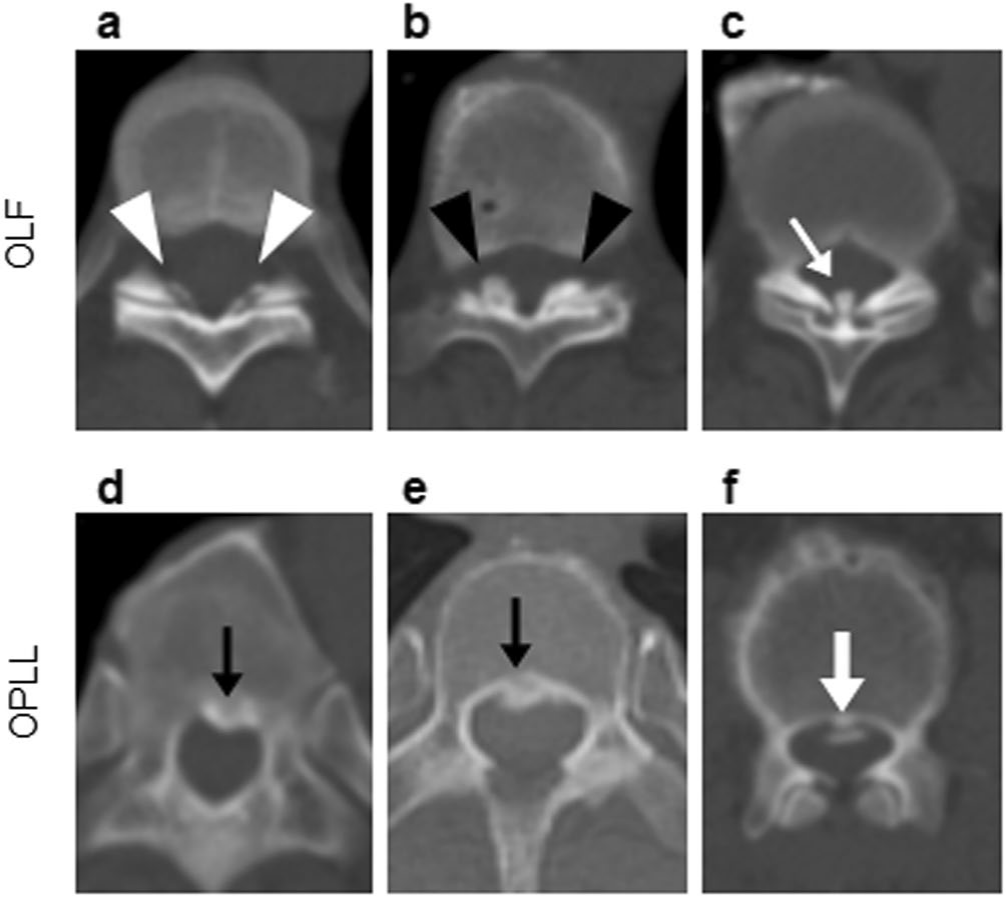
Criteria for the identification of OLF and OPLL on computed tomography. (**a**) OLF that is clearly visible but less than 3 mm in thickness (white arrowheads). (**b**) OLF with an apparent thickness of 3 mm or more (black arrowheads). (**c**) A mushroom-shaped OLF that is localized at the center of the laminae (white arrow). (**d,e**) OPLL with an apparent thickness of 2 mm or more (black arrow). (**f**) OPLL that is small but with a thickness of 2 mm or more (bold white arrow). OLF, ossification of the ligamentum flavum; OPLL, ossification of the posterior longitudinal ligament.

Axial CT images were used to evaluate the distribution of OLF and OPLL (cervical [C], thoracic [T], and/or lumbar [L]). CT was performed using an Aquilion One™/Genesis Edition system (Canon Medical Systems Inc., Tochigi, Japan). The presence of OLF and OPLL was determined according to previous reports (Fig. 2); OLF was essentially defined as ossification of the ligamentum flavum with a thickness of 3 mm or more. Ossification of less than 3 mm, which was clear on axial images, was also considered OLF^3,11^, as was mushroom-shaped ossification localized in the center of the lamina^3,11^. OPLL was essentially defined as ossification of the posterior longitudinal ligament with a thickness of 2 mm or more on axial images^11,29^. All CT scans were assessed by two board-certified spine surgeons; disagreements were resolved by consensus.

### Grouping of subjects

All subjects were divided into the following four groups according to the type of concomitant spinal ligament ossification present: no ligament ossification (control; n = 230), OLF (n = 167), cervical OPLL (C-OPLL; n = 28), thoracic OPLL (T-OPLL; n = 33). Subjects with OPLL in the cervical spine exclusively—with or without concomitant OLF—were classified as the C-OPLL group, and subjects with OPLL in the thoracic spine—with or without concomitant cervical OPLL, lumbar OPLL (L-OPLL), or OLF—were classified as the T-OPLL group. Subjects with OLF without concomitant C-OPLL, T-OPLL, or L-OPLL were classified as the OLF group. This classification was based on the following rationale: (1) C-‍OPLL has a comparatively high probability of occurring concomitantly with OLF^9^; (2) compared to C-OPLL, T-OPLL has distinct features, such as morbid obesity, early onset of symptoms, and diffuse ligament ossification of the entire spine, including OLF, C-OPLL, and L-OPLL^12,15,24^; and (3) OLF occurs mostly in the thoracic spine and rarely in the cervical or lumbar spine^29^.

All subjects were also divided into the following two groups according to the presence or absence of concomitant dyslipidemia: DL(+) (n = 215), and DL(−) (n = 243).

### Statistical analysis

The data were analyzed using BellCurve for Excel (version 3.10; Social Survey Research Information Co., Ltd., Tokyo, Japan). Results are presented as mean ± standard deviation. Four-group comparisons were evaluated using the Kruskal–Wallis and Fisher’s exact tests. Statistical significance was set at *P* < 0.05; however, in the four-group comparisons, it was set at *P* < 0.012 (0.05/4) with a Bonferroni correction considering multiple testing.

Univariate logistic regression analysis was performed for age, body mass index (BMI), sex, comorbidities, the presence of weight gain since the age of 20 years, T-Cho (mg/dL), TG (mg/dL), HDL-C (mg/dL), LDL-C (mg/dL), and the L/H ratio as risk factors for the prevalence of OLF, C-OPLL, and T-OPLL. For variables determined to be significant (*P* < 0.05) in the univariate logistic regression analysis, the relationship between factors affecting the presence of the three types of spinal ligament ossification (i.e., OLF, C-OPLL, and T-OPLL) was evaluated using multivariate logistic regression analysis. Diabetes mellitus was analyzed as a candidate variable regardless of its significance in the univariate logistic regression analysis because it has been mentioned as a risk factor for C-OPLL in the literature. Statistical significance was set at *P* < 0.016 (0.05/3) with a Bonferroni correction considering multiple testing of subjects with multiple concomitant types of ossification.

Cut-off values of the parameters of lipid metabolism for the presence of OLF, C-OPLL, and T-OPLL were calculated from receiver operating characteristic (ROC) curves. In this analysis, only subjects who were not receiving medication were included, considering the effect of medications on serum parameter levels.

## Results

### Baseline patient characteristics

The characteristics of the subjects with OLF and/or OPLL, and the controls without ligament ossification, are shown in Table 1. A total of 49.7% (228/458) had OLF or OPLL; the proportion of subjects with OLF was 36.4% (167/458); C-OPLL, 6.1% (28/458); and T-OPLL, 7.2% (33/458).

**Table 1:** Comparison of clinical characteristics between subjects without ligamentous ossification (control group) and those with OPLL and OLF.

Variable	Control (n = 230)	OPLL + OLF (n = 228)
Age (years)	50.2 ± 9.5	53.6 ± 10.2
**Age range (%)**		
30–39	13.4 (31)	7.0 (16)
40–49	36.0 (83)	30.2 (69)
50–59	32.6 (75)	32.4 (74)
60–69	14.7 (34)	23.2 (53)
70–79	3.0 (7)	7.0 (16)
BMI (kg/m^2^)	23.0 ± 3.3	24.9 ± 3.9
Male (%)	43.0 (99)	66.7 (152)
**Coexisting spinal ligament ossification (%)**		
OLF (not including concomitant OPLL)	0 (0)	72.2 (167)
OPLL (including concomitant OLF)	0 (0)	27.7 (61)
C-OPLL	0 (0)	13.4 (28)
T-OPLL	0 (0)	14.2 (33)
Total	0 (0)	100 (228)

Data are shown as mean ± SD or as percentage (number).

OPLL, ossification of the posterior longitudinal ligament; OLF, ossification of the ligamentum flavum; BMI, body mass index; C, cervical; T, thoracic.

### Association between the ossification types and the proportion of concomitant dyslipidemia

We first examined the comorbidity and severity of dyslipidemia in subjects with each type of spinal ligament ossification. Data on the characteristics of each group are shown in Table 2. The mean BMI of the OLF, C-OPLL, and T-OPLL groups was significantly higher than that of the control group (*P* < 0.01). The mean BMI of the T-OPLL group was the highest among all the groups.

**Table 2:** Comparison of clinical characteristics between subjects without ligamentous ossification (control group) and those with OLF, C-OPLL, and T-OPLL.

Variable	Control (n = 230)	OLF (n = 167)	C-OPLL (n = 28)	T-OPLL (n = 33)
Age (years)	50.2 ± 9.5	53.3 ± 10.5*	54.0 ± 9.6	55.2 ± 9.8*
BMI (kg/m^2^)	23.0 ± 3.3	24.2 ± 3.6*	26.1 ± 3.4*^†^	27.3 ± 4.7*^†^
Male (%)	43.0	64.6*	82.1*	63.6
**Comorbidity (%)**				
Hypertension	14.3	22.1	30.7	33.3*
Diabetes mellitus	2.1	5.3	23.0*^†^	6.0^§^
Ischemic heart disease	5.2	5.9	11.5	12.1
Stroke	1.7	1.7	3.8	0
Renal disease	0.4	0	0	0
Smoking (%)	50.8	58.0	53.8	57.5
Glucose during fasting (mg/dL)	98.3 ± 14.8	103.8 ± 22.9*	110.4 ± 27.1*	105.7 ± 15.5
HbA1c (%)	5.5 ± 0.4	5.7 ± 0.7	6.0 ± 1.0*	5.8 ± 0.5
Weight gain since age 20 years (%)	36.9	50.2*	69.2*	54.5
Exercise for at least 30 min per day (%)	18.2	18.5	11.5	18.1
T-Cho (mg/dL)	210.0 ± 36.5	208.1 ± 31.4	210.4 ± 36.2	204.2 ± 31.7
TG (mg/dL)	91.0 ± 52.8	108.4 ± 62.9*	143.6 ± 81.9*^†^	121.4 ± 51.1*
HDL-C (mg/dL)	65.2 ± 16.0	60.9 ± 15.0*	55.2 ± 14.3*	55.3 ± 13.6*
LDL-C (mg/dL)	122.6 ± 33.3	125.6 ± 30.3	128.7 ± 34.6	126.4 ± 29.9
L/H ratio	2.0 ± 0.8	2.2 ± 0.8	2.4 ± 0.9	2.4 ± 0.9*
L/H ≤ 1.5 (%)	30.4	22.1	15.3	6.0*
L/H ≥ 2.0 (%)	46.0	56.2	69.2	63.6
L/H ≥ 2.5 (%)	25.6	34.1	50.0*	42.4
L/H ≥ 3.0 (%)	13.4	20.9	26.9	30.3

Data are shown as mean ± SD or as percentage. L/H ratio represents LDL-C/HDL-C.

(*P < 0.01 vs. control group).

(^†^*P* < 0.01 vs. OLF group).

(^§^*P* < 0.01 vs. C-OPLL group).

OPLL, ossification of the posterior longitudinal ligament; OLF, ossification of the ligamentum flavum; BMI, body mass index; T-Cho, total cholesterol; TG, triglycerides; HDL-C, high-density lipoprotein cholesterol; LDL-C, low-density lipoprotein cholesterol; C, cervical; T, thoracic.

**Figure 3:**
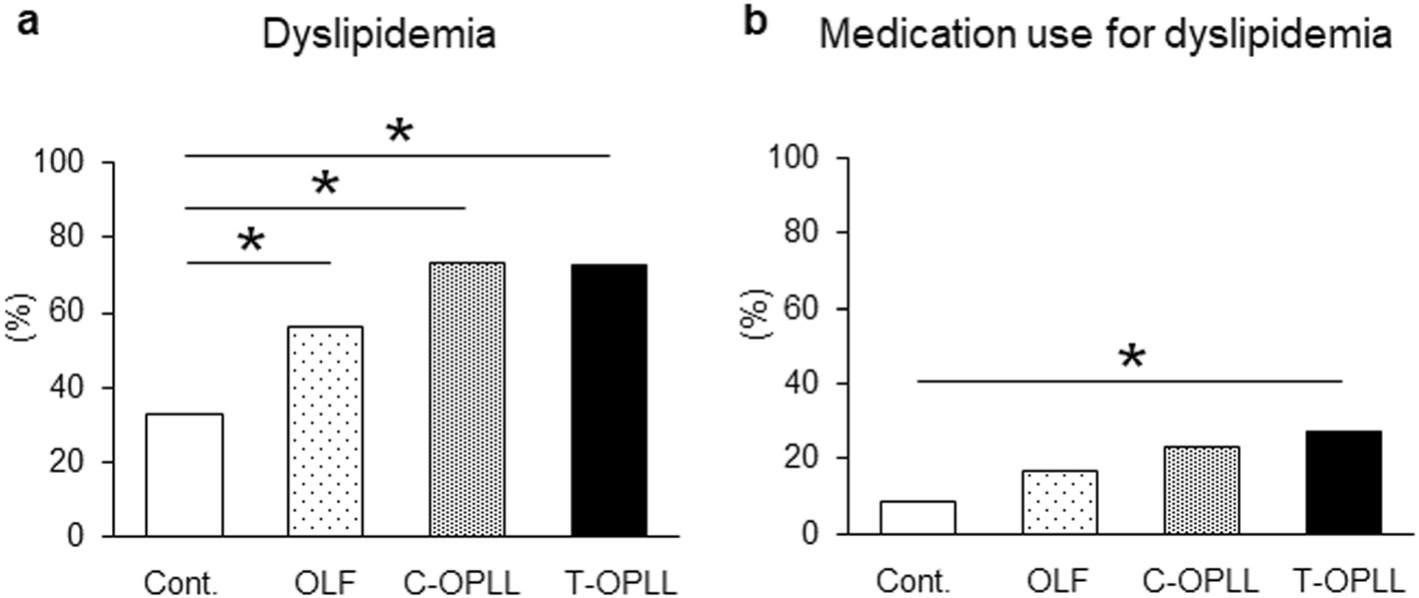
Comparison of the prevalence of dyslipidemia among the ossification types. (**a**) Prevalence of dyslipidemia among the ossification types. (**b**) Rate of the subjects using medication for dyslipidemia among the ossification types. *P < 0.01. OLF, ossification of the ligamentum flavum; OPLL, ossification of the posterior longitudinal ligament; C, cervical; T, thoracic.

The comorbidity of dyslipidemia in the OLF, C-OPLL, and T-OPLL groups was significantly higher than that in the control group (*P* < 0.01). Moreover, the comorbidity of dyslipidemia in the C-OPLL and T-OPLL groups exceeded 70% (control, 33.0%; OLF, 56.2%; C-OPLL, 73.0%, and T-OPLL, 72.7%) (Fig. 3). The mean TG concentration in the OLF, C-OPLL, and T-OPLL groups was significantly higher (*P* < 0.01), while the mean HDL-C concentration was significantly lower (*P* < 0.01), than that of the control group. We also assessed the L/H ratio, one of the indicators of the severity of dyslipidemia. The proportion of subjects with an L/H ratio ≤ 1.5 in the T-OPLL group was significantly lower than that in the control group (*P* < 0.01). The proportion of subjects with an L/H ratio ≥ 3.0 in the OLF, C-OPLL, and T-OPLL groups was higher than that in the control group, while the proportion in the T-OPLL group was the highest; however, the differences were not significant.

### Rate of subjects with concomitant spinal ligament ossification according to the presence of dyslipidemia

Baseline clinical information and the results of lipid-related parameters based on blood sampling are shown in Table 3. The mean age, BMI, male ratio, and proportion of concomitant hypertension and diabetes mellitus in the DL(+) group were significantly higher than those in the DL(−) group. Furthermore, the proportion of subjects without ligament ossification in the DL(+) group was significantly lower than that in the DL(−) group (*P* < 0.001), whereas the comorbidity of OLF, C-OPLL, and T-OPLL in the DL(+) group was significantly higher than that in the DL(−) group (*P* < 0.01).

**Table 3:** Comparison of clinical characteristics between subjects with dyslipidemia (DL[+]) and those without dyslipidemia (DL[−]).

Variable	DL( −) (n = 243)	DL( +) (n = 215)	*P* value
Age (years)	50.5 ± 10.2	53.5 ± 9.6	< 0.001
BMI (kg/m^2^)	22.8 ± 3.5	25.3 ± 3.6	< 0.001
Male (%)	44.8	66.0	< 0.001
**Comorbidity (%)**			
Hypertension	16.0	23.7	0.03
Diabetes mellitus	2.4	7.9	0.007
Ischemic heart disease	5.3	7.4	0.36
Stroke	1.6	1.8	0.86
Renal disease	0.4	0	0.34
T-Cho (mg/dL)	196.1 ± 28.0	223.4 ± 35.0	< 0.001
TG (mg/dL)	71.0 ± 30.4	138.6 ± 65.2	< 0.001
HDL-C (mg/dL)	68.7 ± 15.3	55.1 ± 12.7	< 0.001
LDL-C (mg/dL)	108.2 ± 23.0	142.3 ± 31.2	
**Coexisting spinal ligament ossification (%)**			
No ligament ossification	63.3	35.3	< 0.001
OLF (not including concomitant OPLL)	29.6	43.7	0.001
OPLL (including concomitant OLF)	6.9	25.5	< 0.001
C-OPLL	3.2	14.4	< 0.001
T-OPLL	3.7	11.1	0.002

Data are shown as mean ± SD or as percentage.

DL, dyslipidemia; OPLL, ossification of the posterior longitudinal ligament; OLF, ossification of the ligamentum flavum; BMI, body mass index; T-Cho, total cholesterol; TG, triglycerides; HDL-C, high-density lipoprotein cholesterol; LDL-C, low-density lipoprotein cholesterol; C, cervical; T, thoracic.

### Risk factors for the prevalence of OLF, C-OPLL, and T-OPLL

**Figure 4:**
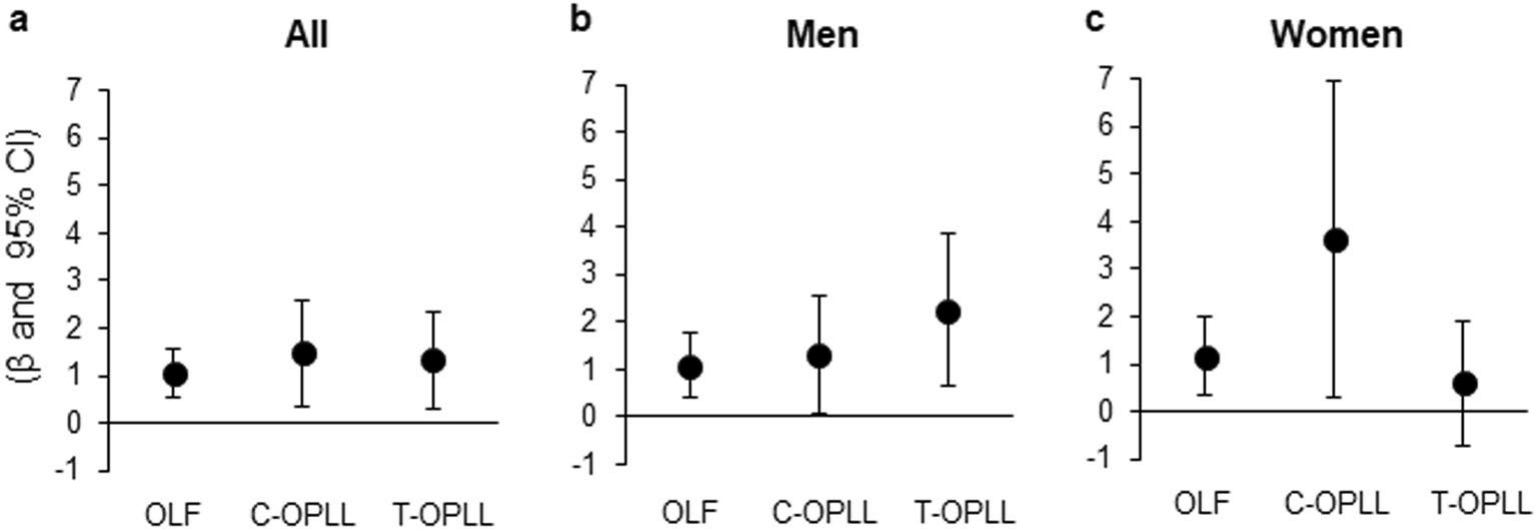
Comparison of the correlation of dyslipidemia with OLF, C-OPLL, and T-OPLL. Results shown are β-values with 95% CIs (error bars). (**a**) All subjects. (**b**) Men. (**c**) Women. OLF, ossification of the ligamentum flavum; OPLL, ossification of the posterior longitudinal ligament; β, regression coefficient; CI, confidence interval; C, cervical; T, thoracic.

After characterizing the comorbid ossification types associated with dyslipidemia, we conducted a multivariate logistic regression analysis to identify risk factors for an increased prevalence of OLF and OPLL. Risk factors for the presence of OLF, C-OPLL, and T-OPLL, respectively, were compared to subjects without spinal ligament ossification. (i.e., a maximum of three duplicate analyses were conducted for subjects who had all three types of concomitant ossification). The regression coefficients (β) and standardized β-values are shown in Table 4 and Fig. 4. Dyslipidemia was found to be associated with the prevalence of OLF, C-OPLL, and T-OPLL. Comparing the β-values among the ossification types, C-OPLL was the most strongly associated with dyslipidemia (β, 1.48; 95% CI 0.35–2.60). On analysis of risk factors by sex, T-OPLL was the most strongly associated with dyslipidemia in men (β, 2.24; 95% CI 0.65–3.84), whereas C-OPLL was the most strongly associated with dyslipidemia in women (β, 3.64; 95% CI 0.31–6.97). Furthermore, age was associated with the prevalence of T-OPLL, being male with the prevalence of OLF and C-OPLL, and BMI with the prevalence of OLF and T-OPLL. Diabetes mellitus and the L/H ratio was not associated with the prevalence of any ossification type.

**Table 4:** Multivariate logistic regression analysis of risk factors for the prevalence of spinal ligament ossification compared to subjects without spinal ligament ossification.

Type of ossification	Independent variables	β	Standardized β	95% CI	*P* value
OLF	Dyslipidemia	1.03	0.51	0.52–1.54	< 0.001
	Male	0.73	0.36	0.30–1.17	< 0.001
	BMI (kg/m^2^)	0.08	0.31	0.02–0.14	0.006
	Age (years)	0.02	0.24	0.00–0.04	0.018
	L/H ratio	− 0.27	− 0.23	− 0.58 to 0.03	0.07
	Diabetes mellitus	0.37	0.08	− 0.68 to 1.44	0.48
C-OPLL	Dyslipidemia	1.48	0.71	0.35–2.60	0.010
	Male	1.43	0.71	0.32–2.53	0.011
	BMI (kg/m^2^)	0.15	0.55	0.01–0.30	0.03
	Diabetes mellitus	1.68	0.35	0.28–3.08	0.018
	Age (years)	0.01	0.18	− 0.02 to 0.06	0.41
	L/H ratio	− 0.20	− 0.17	− 0.79 to 0.38	0.49
T-OPLL	BMI (kg/m^2^)	0.28	1.09	0.16–0.40	< 0.001
	Dyslipidemia	1.32	0.64	0.30–2.34	0.011
	Age (years)	0.05	0.53	0.01–0.09	0.013
	L/H ratio	− 0.26	− 0.22	− 0.81 to 0.28	0.34
	Diabetes mellitus	− 1.09	− 0.17	− 3.07 to 0.88	0.27
	Male	0.29	0.14	− 0.60 to 1.19	0.27

β, regression coefficient; CI, confidence interval; OPLL, ossification of the posterior longitudinal ligament; OLF, ossification of the ligamentum flavum; BMI, body mass index; C, cervical; T, thoracic.

To determine the relative contribution of risk factors to ossification, we compared the standardized β-values for each type of spinal ligament ossification. For both OLF and C-‍OPLL, the standardized β-value for dyslipidemia was higher than that for age, BMI, being male, the L/H ratio, and diabetes mellitus. For T-OPLL, the standardized β-value for BMI was higher than that for dyslipidemia.

Finally, we calculated the cut-off values of the parameters of lipid metabolism for the presence of OLF, C-OPLL, and T-OPLL by the ROC curves. For C-OPLL, TG was 104 mg/dL (area under the ROC curve [AUC], 0.71; sensitivity, 0.80; specificity, 0.71) and HDL-C was 57 mg/dL (AUC, 0.70; sensitivity, 0.66; specificity, 0.65) (Supplemental Table 1).

## Discussion

The present study showed that the prevalence of dyslipidemia in subjects with OLF and OPLL was higher than we expected (56–73%), and was 1.6–2.2 times higher than that of subjects without ligament ossification (33%). The clinical significance of this finding is that dyslipidemia in OLF and OPLL was identified using a large dataset, which has not been previously focused on. Considering that this study included a large number of healthy community residents and institutional staff, the possible reason for the high prevalence of dyslipidemia in these subjects may involve metabolic disturbances related to visceral fat—such as obesity and MAFLD—rather than a sedentary lifestyle, physical inactivity, and sarcopenia, which are related symptoms of myelopathy. Because OLF and OPLL often coexist^9–12^, risk factors may have been confounded in the past; however, we distinguished between ossification types and successfully analyzed the association with dyslipidemia.

Our results suggest that dyslipidemia is associated with the development of spinal ligament ossification. This was reinforced by the (1) increased proportion of subjects with higher L/H ratios in OLF and OPLL; (2) higher proportion of these conditions in the DL(+) than the DL(−) group; and (3) results of the multivariate logistic regression analysis that correlated the prevalence of these conditions with dyslipidemia. An important finding was that dyslipidemia was more strongly associated with the prevalence of OLF and C-‍OPLL than with other risk factors when compared using standardized β-values for the relative risk of each ossification type. We also determined the relative strength of the association of dyslipidemia with the prevalence of OLF, C-OPLL, and T-OPLL by comparing β-values. Interestingly, dyslipidemia was most strongly associated with the prevalence of C-OPLL in women. These results may reflect the fact that some patients with symptomatic OLF and those with C-OPLL have a BMI exceeding the average BMI of the general Japanese population, and that patients with T-OPLL tend to have severe obesity (BMI ≥ 35 kg/m^2^). Recently, it has been reported that the severity of ossification of the entire spine in patients with symptomatic OPLL is associated with the severity of MAFLD rather than being associated with BMI^24^. The fact that patients with OPLL have a high frequency of comorbid lifestyle-related diseases also suggests that ossified lesions may be exacerbated by abnormal lipid metabolism related to visceral fat obesity, rather than by mere local instability or mechanical stimulation^30–32^.

The mechanisms by which abnormal lipid metabolism causes ectopic ossification of spinal ligaments are speculative. (1) Visceral fat accumulation and fatty liver promote the production of LDL-C in the liver^17–19^. (2) The oxidative stress environment caused by hyper-LDLemia activates Wnt3 secretion from vascular endothelial cells and Wnt signaling via LDL receptor-related protein 5 (LRP5) or LRP6, which induce atherosclerotic calcification^20–22^. (3) In bone, LRP5/Wnt/Frizzled forms a complex^21,22^. This signaling activity results in the accumulation of beta-catenin, which regulates the expression of many genes, including Cbfa-1, which is essential for osteoblast differentiation^20–22^. The details of the relationship between abnormal lipid metabolism and ossification of spinal ligaments should be studied in the future.

By means of multivariate logistic regression analysis, we identified further independent risk factors for spinal ligament ossification: BMI for OPLL and T-OPLL; being male for OLF and C-OPLL; and age for T-OPLL. Obesity is more common in symptomatic OLF and T-OPLL, with a strong ossification tendency of the entire spinal ligament^12,13^. In addition, OLF and C-OPLL are more likely to occur in men^6,29^, whereas T-OPLL is more likely to occur in women^5^. Our results confirm the previous findings; however, we would like to emphasize that our results are not necessarily synonymous with the risk of worsening ossified lesions, because the present study was based on a risk analysis of an increased prevalence of OLF and OPLL, and included many asymptomatic subjects with relatively small ossified lesions.

The prevalence of OLF (36.4%), C-OPLL (6.1%), and T-OPLL (7.2%) should be interpreted with caution because subjects with only ossification of the anterior longitudinal ligament (OALL) were not included in this study. In our previous study with a similar approach, asymptomatic subjects with only OALL were about 14%^11^. In previous large CT-based studies of Japanese subjects, the prevalence of OLF was 36.3%, C-OPLL was 6.3%, and T-OPLL was 1.6–1.9%^3,5,29^; the prevalence of OLF and C-OPLL was comparable to our results. The reason why the prevalence of T-OPLL was different from previous studies was probably due to the different definition of T-OPLL in this study compared to previous ones.

There are several limitations to the present study. First, it has a cross-sectional design and it is not possible to conclude whether dyslipidemia is a causative factor or a consequence of OLF or OPLL. Second, we used a large single-center database, but the sample size of the C-OPLL and T-OPLL groups was small. Given that the prevalence of OPLL in the general Japanese population is low^5–7^, a multicenter study is needed to validate our results. Third, the present study is based on data from the Japanese population, which may not necessarily be applicable to other populations. Forth, all subjects underwent neck-to-pelvis CT by their own choice, although the majority of the 12,740 subjects who underwent a physical examination did not undergo CT, or underwent either neck-to-chest or abdomen-to-pelvis CT. Thus, the risk of selection bias cannot be completely excluded. Finally, we did not compare the size of OPLL/OLF with specific values of lipid abnormalities.

In summary, this study found that dyslipidemia is a risk factor for an increased prevalence of OLF, C-OPLL, and T-OPLL in Japanese individuals. Dyslipidemia was more strongly associated with spinal ligament ossification than other risk factors, especially in OLF and C-OPLL. Our results suggest that dyslipidemia is not only notably associated with the development of spinal ligament ossification, but that it also bears equal, if not higher, importance to other previously noted lifestyle-related diseases and obesity. Further studies are needed to clarify whether dyslipidemia is a causative factor for spinal ligament ossification.

## Supplementary Information

Supplementary Table 1.

## Data Availability

The datasets generated and/or analyzed during the current study are not publicly available due to the nature of this research, participants of this study did not agree for their data to be shared publicly, but are available from the corresponding author on reasonable request.
