# Supplementary material for: Strong relationship between dyslipidemia and the ectopic ossification of the spinal ligaments
Source: Sci Rep. 2022 Dec 30;12:22617. doi: 10.1038/s41598-022-27136-4 (PMC9803662; doi:10.1038/s41598-022-27136-4)
Supplement: Supplementary file 1 — Supplementary Table 1. [file 41598_2022_27136_MOESM1_ESM.docx]

| **Supplemental table 1.** Cut-off values for lipid abnormalities on the prevalence of spinal ligament ossification by ROC curve | | | | | | |
| --- | --- | --- | --- | --- | --- | --- |
| **Type of ossification** | **Variables** | **Cut-off** | **AUC** | **Sensitivity** | **Specificity** |  |
| OLF | TG (mg/dL) | 98 | 0.59 | 0.50 | 0.70 |  |
|  | HDL-C (mg/dL) | 59 | 0.58 | 0.52 | 0.63 |  |
|  | LDL-C (mg/dL) | 128 | 0.53 | 0.49 | 0.56 |  |
|  | L/H ratio | 2.0 | 0.58 | 0.58 | 0.57 |  |
| C-OPLL | TG (mg/dL) | 104 | 0.71 | 0.80 | 0.71 |  |
|  | HDL-C (mg/dL) | 57 | 0.70 | 0.66 | 0.65 |  |
|  | LDL-C (mg/dL) | 127 | 0.57 | 0.66 | 0.54 |  |
|  | L/H ratio | 2.3 | 0.68 | 0.66 | 0.71 |  |
| T-OPLL | TG (mg/dL) | 114 | 0.67 | 0.62 | 0.76 |  |
|  | HDL-C (mg/dL) | 53 | 0.68 | 0.54 | 0.76 |  |
|  | LDL-C (mg/dL) | 134 | 0.59 | 0.58 | 0.64 |  |
|  | L/H ratio | 2.2 | 0.67 | 0.62 | 0.68 |  |
| ROC, receiver operating characteristic; AUC, area under the ROC curve; OPLL, ossification of the posterior longitudinal ligament; OLF, ossification of the ligamentum flavum; TG, triglycerides; HDL-C, high-density lipoprotein cholesterol; LDL-C, low-density lipoprotein cholesterol; C, cervical; T, thoracic | | | | | | |
